# Incidence, causes, and consequences of preventable adverse drug events: protocol for an overview of reviews

**DOI:** 10.1186/s13643-016-0392-4

**Published:** 2016-12-05

**Authors:** Brian Hutton, Salmaan Kanji, Erika McDonald, Fatemeh Yazdi, Dianna Wolfe, Kednapa Thavorn, Sally Pepper, Laurie Chapman, Becky Skidmore, David Moher

**Affiliations:** 1 Ottawa Hospital Research Institute, Ottawa, ON K1H 8L6 Canada; 2 School of Epidemiology, Public Health and Preventive Medicine, University of Ottawa, Ottawa, Canada; 3 The Ottawa Hospital, Ottawa, Canada; 4 Institute of Clinical and Evaluative Sciences (ICES uOttawa), Ottawa, Canada; 5 Health Canada, Marketed Health Products Directorate, Ottawa, Canada

**Keywords:** Systematic review, Overview of reviews, Adverse drug reactions, Adverse drug events, Medication errors

## Abstract

**Background:**

Medication errors represent a noteworthy source of harm to patients. In recent years, several systematic reviews have assessed the frequency and causes of these events, as well as other factors such as commonly associated drugs, their incidence in different specialties, and their consequences to patients. Despite this past literature, there remains a need to study discrepancies between these reviews and establish the current state of the evidence. The planned review will bring together, compare, and contract existing evidence related to the occurrence of medication errors in acute and continuing/long-term care settings.

**Methods:**

A systematic review of reviews will be performed. A literature search designed by an experienced information specialist will be carried out in Medline, Embase, and the Cochrane Library. We will seek systematic reviews and meta-analyses of primary research studies that evaluate one or more of the following aspects of the occurrence of preventable adverse drug events in hospitals and long-term care centers: the incidence of preventable adverse drug events, either overall or within subgroups of interest related to setting; drug or patient characteristics; cited consequences of these events to patients, including death, emergency room visits, or other outcomes; and established causes of the preventable adverse drug events. Two researchers will independently screen all abstracts and full texts for study selection and subsequently perform data extraction from all included studies. Quality of the reviews will be assessed using the assessing the methodological quality of systematic reviews (AMSTAR) tool. Where objectives from two or more reviews overlap, we will employ the Jadad framework to assess the causes of any noted discrepancies between reviews. An overall summary of results will be performed using tabular and graphical approaches and will be supplemented by narrative description.

**Discussion:**

This overview will help synthesize the broad degree of information available on this important topic. This review is being performed by members of the Drug Safety and Effectiveness Network along with collaboration from Health Canada, and its findings will be published in a peer-reviewed journal. The results may also inform future research in this area.

**Systematic review registration:**

PROSPERO CRD42016043220

**Electronic supplementary material:**

The online version of this article (doi:10.1186/s13643-016-0392-4) contains supplementary material, which is available to authorized users.

## Background

Medical errors are the third leading cause of death in the USA, only behind heart disease and cancer [1]. The 2004 Canadian Adverse Events Study reported an incidence of 70,000 preventable adverse events per 2.5 million annual hospitalizations in Canada. Investigators estimated that between 9250 and 23,750 deaths due to adverse events could have been prevented in the year 2000 alone [2]. Although the Canadian Adverse Events Study identified that 24% of adverse events were due to medications [2], the study was not designed to capture medication errors specifically; these are often not well documented and can be difficult to detect. For example, errors due to inappropriate use or inadequate monitoring likely would not have been captured [3].

These types of pivotal studies highlight an opportunity to improve care provided to patients while additionally reducing the burden on our healthcare system. Development of an enhanced understanding of *why*, *how*, *when*, and *where* preventable adverse events occur could inform potential interventions to reduce the risk of their occurrence. Because medications are involved in almost a quarter of all adverse events, this represents a logical first target in addressing this issue. Preliminary scoping of the literature in this area suggests that there exist a number of review articles that have sought to characterize the prevalence of adverse drug events in different populations and settings [4–11]. Some of the previous research incorporated estimation of the proportion of preventable adverse drug events. However, an assessment of research methods and discussions among academic researchers highlighted the existence of confusion regarding terminology such as adverse drug events, adverse drug reactions, medication errors, and medication incidents [12]. Variations in endpoint definitions and preventability criteria are likely to be associated with differences in findings across published studies.

The Institute for Safe Medication Practices (ISMP) Canada defines an adverse drug event as “An injury from a medicine or lack of an intended medicine” and “includes adverse drug reactions and harm from medication incidents [13].” An adverse drug reaction is defined by the World Health Organization as “any response to a drug which is noxious and unintended which occurs at doses normally used in man for prophylaxis, diagnosis, or therapy of disease, or for the modifications of physiological function [14]” and is a type of adverse drug event by these definitions. The occurrence of a preventable adverse drug event implies that an injury or harm occurred as a result of a medication error, which is further defined as “a failure in the treatment process that leads to, or has the potential to lead to, harm to the patient [15].” In contrast, ISMP refers to this as a “medication incident [13].”

Currently, Health Canada’s guidance with respect to adverse reaction reporting indicates that all suspected adverse drug reactions should be reported, especially if they are associated with drugs newly marketed within the past 5 years, are unexpected, or are serious [16]. Adverse reactions are defined broadly by Health Canada as a “noxious and unintended effect to health products [16].” The passing of “Vanessa’s law” in 2014 will require that all serious adverse drug reactions are reported by healthcare institutions; however, the accompanying regulations are still in development.

In theory, mandatory reporting of serious adverse drug reactions by all health care practitioners could be an important step in improving post-market surveillance. However, mandatory reporting would represent a major change in practice, increased workload for health professionals, and potential increased cost to the health care system. Reporting rates have been shown to be low even in countries such as New Zealand where there has been “mandatory” reporting of adverse drug reactions by health professionals. Thus, enforcing such a requirement would be challenging [17].

Our research team is interested in further developing an understanding of the magnitude of preventable adverse drug events within the broader context of pharmacovigilance, with the notion that such knowledge may prove useful to inform modifications of current post-market surveillance activities. Preliminary searching of the literature suggests there are a variety of existing systematic reviews that study the incidence of preventable adverse drug events. In such situations, an overview of reviews [18, 19] is a logical study design, affording the research team the opportunity for (a) comparison of review findings to establish their degree of consistency or differences when their objectives overlap, as well as assessment of differences in methods (e.g., inclusion criteria, analytic plan, or other aspects of study rigor), included studies, outcomes assessed, and other factors which may be associated with any observed differences in findings; (b) compilation of complementary but distinct information from different reviews to be brought into one source; and (c) identification of gaps in terms of outcomes of importance not yet studied or important variations across reviews in those that have been studied.

An overview of reviews (i.e., a systematic review of systematic reviews) will enable us to map and compare the objectives, methods, and findings of existing systematic reviews related to this topic to develop a greater understanding of the information available regarding the incidence of preventable adverse drug events, common factors contributing to preventable adverse drug events, and other considerations. These reviews may have relevant/related but different objectives and include a variety of study types and variations in study methods and characteristics which can provide data related to the objectives of the review.

## Study methods

This protocol describes the methods to be followed for conduct of the planned overview of reviews. Methods have been chosen in consultation with the Cochrane Handbook’s chapter on methods for overviews of reviews [18], as well as recent work by Smith [19]. As the objective of the review will be to compare and contrast findings regarding measures of preventable adverse drug events across reviews, Jadad’s framework for assessment of discordant reviews will also be used [20]. This review has been registered in the PROSPERO database (CRD42016043220), and this protocol has been prepared in consultation with the PRISMA-P statement [21, 22] (checklist provided in the supplemental documents to this protocol, in Additional file 1). Any amendments to the protocol shall be described in the final review.

### Research questions to be addressed

This study has been designed to answer the following primary research question: *What is the incidence of preventable adverse drug events in acute and continuing/long-term care hospitals/institutions (including both academic and community hospitals)?* In the context of the review, a series of secondary objectives will also be addressed. These will include assessment of PADR incidence within different patient age groups (e.g., pediatric, adult, and elderly patients), settings (e.g., acute, continuing, and long-term care; academic vs community hospitals), and clinical specialties (e.g., emergency medicine, cardiology); assessment of the different cited types and causes of preventable adverse drug events (both with their corresponding distribution of frequency); and the severity of patient outcomes associated with their occurrence.

### Study eligibility criteria

Eligibility criteria have been prepared in terms of the population-intervention/comparator-outcomes-study design (PICOS) framework.

#### Population

Studies involving patients receiving acute or ambulatory care from hospitals and being treated with drug therapy will be sought. Studies in other institutional settings such as long-term care facilities will also be included. Studies related to primary care will not be eligible. However, data coming from inpatient vs outpatient settings as well as hospital-based vs other settings will be eligible.

#### Intervention/comparators

No specific intervention is required for a study to be eligible for this overview of reviews; studies reporting eligible patient data and patient outcomes will be included. Studies with some aspect of causality between a drug and an event are of primary interest though those studies without a clear causal association are also of interest.

#### Outcomes

A specific definition for *preventable adverse drug event* will not be established as an a priori eligibility criteria. Definitions are likely to vary by study and may be highly associated with variations in results. As such, studies with variable definitions will be included, and any discrepancies in results associated with different definitions will be formally assessed. Reviews that include studies dealing with ameliorable adverse drug events (i.e., those that could not have been prevented but whose severity could have been limited) or studies related to interactions with herbal or non-prescription medications will be included. Those reporting on the occurrence of adverse drug events where medications were adequately administered will not be eligible. With regard to measures of interest for the current review, studies will be included if they have addressed one or more measures among incidence of preventable adverse drug events, distribution of causes observed (including miscommunicated drug orders, drug packaging/labeling problems, drug storage and delivery problems, lack of staff or patient education, lack of quality check mechanism, and other forms of cause), patient consequences (including no harms incurred, hospitalization incidence, requirement for life-saving interventions, emergency room visit, death, quality of life changes, and other consequences), and evaluations of severity. Incidence of preventable adverse drug events will be the primary outcome of interest, while all others will be considered secondary outcomes.

#### Study design

The current study is designed to be an overview of existing reviews, and thus, eligible study designs will be restricted to those which have employed a systematic review design. We will consider publications to be systematic reviews if they were clearly described in the report as being based on a systematic search of one or more electronic literature databases, clearly specified the review question and eligibility criteria, involved study selection and data collection by two or more reviewers, performed some form of risk of bias appraisal of included studies, and synthesized all information using a quantitative or qualitative approach. Review articles not meeting these criteria will be excluded.

### Searching the literature for relevant studies

A search strategy has been established with the participation of an experienced information specialist (BS) and independently peer-reviewed by a second information specialist using PRESS criteria (Additional file 2) [23]. We will search Medline, Embase, and the Cochrane Library without date restrictions. Key search terms will include the following: *adverse drug reaction reporting systems*, *drug-related side effects and adverse reactions*, *medication errors*, and an in-depth list of text words given the nature of varying terminology in this area. The search will be restricted to publications in English and French.

### Process of study selection

Two reviewers will independently screen all citations identified from the literature search described above. All citations judged potentially eligible will be screened in full text; any disagreements will be settled by consultation of a third member of the review team. An analogous process will be used to screen full texts. Both stages of screening will be preceded by a piloting exercise to ensure that reviewers have a similar understanding of the eligibility criteria. As recommended by the PRISMA Statement [24], a flow diagram will be presented to describe the process of study selection. Management of all screening will be performed using the Distiller SR software (Evidence Partners Inc., Ottawa, Canada).

### Data extraction and risk of bias assessment

Given that we will seek to address multiple objectives, a broad range of information will be collected from each of the included studies to derive all relevant data. This will include each of the following items described in Table 1; it should be noted that for many of these items, subsequent synthesis of findings will involve summarizing/contrasting these features in relation to the primary studies that each review assessed. Data collection will be performed by two reviewers independently, with involvement of a third reviewer as needed to establish consensus in the presence of disagreements. A data collection form will be drafted and piloted on a small number of studies and discussed among the team to incorporate any necessary refinements prior to completion of data collection from all relevant studies. Methodological quality/risk of bias for included systematic reviews will be performed using the AMSTAR tool [25]; this tool will help to distinguish the rigor of included reviews in terms of methods used for literature searching, processes for study selection and data collection, data analysis, and other vital aspects of systematic review design. A table will be presented displaying the scoring of AMSTAR item assessments for each included review, which will enable comparison of the rigor and reporting of included reviews.

**Table 1:** Elements for data collection

Aspect of data collection	Description of key items to be extracted
General review characteristics and patient traits	Primary details gathered will include • Stated review objectives • Numbers of included studies and patients • Date ranges of literature searches • Study eligibility criteria • Patients’ age distribution (pediatric vs adult vs elderly) • Diagnoses/comorbidities of patients studied (number and/or cluster of comorbid condition) • Any other key patient/population information that were noted in the review
Hospital/setting characteristics of each review’s included studies	Key setting-related factors for each review’s included studies. Such details will include • Hospital size characterized by the number of beds and/or the number of staff • Academic vs community centers • Clinical disciplines involved in the primary studies • Geographic locations of the primary studies • Any other relevant features presented in each review regarding its primary studies
Quantitative measures of incidence of preventable adverse drug events	Outcome data collected will include • Stated eligible measures/definitions of the endpoints assessed in each eligible review, including preventable adverse drugs events and other endpoints related to the current review objectives • Corresponding incidence rates of primary studies in each review’s included studies (and any presented meta-analysis results thereof, both overall or within specific subgroups of patients) • Severity assessments as presented for each review’s set of included studies (if reported) • Causality or likelihood assessments as presented for each review’s set of included studies (if reported) • Method of collection of adverse event data (i.e., spontaneous vs active surveillance) for each review’s set of included studies (if reported)
Causes of preventable adverse drug events identified among each review’s included studies	Causes of interest will include (but not be limited to) the following • Miscommunications of drug order • Drug name, label, or packaging problem • Drug storage or delivery problem • Environmental, staffing, or workflow problem • Staff or patient education problem • Lack of quality control or independent check systems • Problem with electronic data management system
Consequences of adverse drug events	Consequences of interest will include (but not be limited to) proportions of preventable adverse drug events with the following consequences • No harm done • Prolonged hospitalization needed • Life-saving treatment needed • Hospital readmission • Emergency department visit readmission • Transient impact • Permanent impact • Quality of life • Death • Other outcomes reported
Incidence and types of preventable adverse drug events reported within key subgroups of interest	Subgroups of interest include (but are not limited to) • Clinical specialty (cardiology, oncology, etc.) • Patient age (e.g., pediatric, adults, elderly) • Presence of comorbidities • Healthcare setting

All data collection will be performed electronically using the Microsoft Excel software (Microsoft Corporation, Seattle, USA).

### Synthesis of data

To summarize findings, a descriptive approach will be taken that will include tables to characterize key features, findings, and variations of the research, supplemented with graphics to highlight diversity in study results and other aspects; guidance from the Cochrane Handbook for overviews of reviews will be followed. This will also include a focused effort to map gaps between reviews in relation to many aspects of preventable adverse drug events described. The Jadad framework for discordant reviews will also be used to assess variations in reviews [20]. For reviews addressing the same objectives and endpoints, their findings will be compared.

In exploring the rationale for variations in findings between the reviews, several strategies will be employed. Firstly, comparison of review methods will be performed in relation to eligibility criteria (i.e., assessment of variations in criteria used to identify eligible patients, study designs, and endpoints of interest), literature search details (dates, databases, and key differences in strategies employed; language restrictions employed), endpoint definitions used, statistical approaches to meta-analysis (if performed), and rigor of review methods (as reflected by variations in AMSTAR assessments and other aspects of study methodology). Secondly, the evidence base included in different reviews will be evaluated in terms of their degree of overlap; this will involve comparison of date ranges of studies covered by the review, the numbers of studies and patients across reviews and development of a citation matrix to establish the similarity of included study lists. Lastly, comparison of review findings (e.g., meta-analytic findings regarding pooled incidence rates or other related measures) and conclusions drawn by review teams will also be performed.

### Reporting of review findings

To present findings from our review, we will draft a summary manuscript for publication in a peer-reviewed, open-access journal. We will follow recommendations from the PRISMA Statement to guide the reporting of this work [24], and will include a PRISMA Checklist to document completeness of reporting.

## Discussion

Adverse events related to drugs have been established as having notable associations with the occurrence of morbidity in patients. Numerous past reviews have addressed different aspects of questions surrounding the incidence, causes, and consequences of preventable adverse drug events. There is a need to map the findings from these reviews to enhance our understanding of all the data currently available, as well as to identify discrepancies in findings and resolve their sources. These are all intended goals for the planned overview of reviews. Achieving these objectives will prove helpful in establishing improved efforts to reduce the frequency of adverse drug events, both overall and in terms of the occurrence of events with particularly severe consequences for patients.

We anticipate certain challenges during the conduct of the review. These may include variation across reviews in definitions of preventability (e.g., “inappropriate use” vs obvious error of omission); methods of measurement of preventability; definitions of adverse drug event, adverse drug reaction, medication error, and corresponding variations in methods for measurement; approaches used to assess the likelihood of causality; jurisdictional differences in the reporting of adverse drug events; and settings, populations, or population characteristics (i.e., inpatient service/area, type of hospital, age, comorbidities, complexity, number of medications, etc.). Using rigorous data collection and a systematic approach to evaluation of endpoints in relation to study characteristics, we will carefully address these challenges while summarizing the existing body of literature to establish the current state of the evidence and to identify remaining gaps. We will publish our findings with the goal of helping to inform potential research directions and  changes in post-market surveillance in the future.

## Additional files

Additional file 1: PRISMA-P Checklist. (DOC 71 kb)
Additional file 2: Medline Search Strategy. (DOC 32 kb)

## Abbreviations

AMSTAR: Assessing the methodological quality of systematic reviews
ISMP: Institute for safe medication practices
PADR: Preventable adverse drug reactions
PICOS: Population-intervention/comparator-outcomes-study design
PRISMA: Preferred reporting items for systematic reviews and meta-analyses
